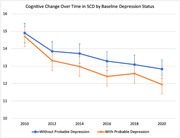# Depressive Symptoms and Cognitive Function in Older Adults with Subjective Cognitive Decline: Longitudinal Findings From 2010 to 2020

**DOI:** 10.1002/alz.090034

**Published:** 2025-01-09

**Authors:** Jing Huang, Nancy Perrin, Junxin Li

**Affiliations:** ^1^ Johns Hopkins University, Baltimore, MD USA

## Abstract

**Background:**

Subjective cognitive decline (SCD) is increasingly recognized as a symptom of preclinical Alzheimer’s disease (AD), characterized by self‐perceived cognitive worsening without objective cognitive deficits. Depressive symptoms have been linked to elevated risks of cognitive decline, yet research on depression’s role in SCD progression is still limited and inconsistent. Therefore, this study aims to examine the longitudinal association between depressive symptoms and cognitive function in older adults with SCD over a 10‐year period.

**Method:**

Using six waves of biennial data from the Health and Retirement Study (2010‐2020), we selected older adults aged 50+ with SCD in 2010. SCD was defined by self‐reported memory decline compared with 2 years ago, absence of objective cognitive deficits (Modified Telephone Interview for Cognitive Status [TICS‐M]>11), and no self‐reported AD or dementia. Linear mixed models estimated the association between baseline depressive symptoms and cognitive function change from 2010 to 2020. Time, depression, and the time by depression interaction predicted cognitive function, measured by TICS‐M. Depressive symptoms were assessed by the eight‐item Center for Epidemiologic Studies Depression Scale (CESD‐8). CESD‐8 scores (0 – 8) were dichotomized into with and without depression using a cutoff of 3. Covariates included age, sex, race, education, marital status, poverty level, smoking, drinking, sleep disturbance, and number of chronic conditions.

**Result:**

3058 older adults with SCD were identified (65.8 ± 10.5 years, 65.3% females). The baseline CESD score averaged 2.03 (SD = 2.32), and 31% were categorized as having probable depression. The mean TICS‐M score was 16.07 (SD = 3.00) in 2010, declining to 14.71 (SD = 4.28) in 2020. Compared with individuals without depression, those with depression at baseline exhibited a significantly greater decline in cognitive function over time when controlling for covariates (*p* = 0.001). Those with depression showed an average 0.14 greater decline in cognitive function at each biennial wave compared to those without depression (depression × time coefficient = ‐0.14, 95% CI = ‐0.22, ‐0.06).

**Conclusion:**

This study provides longitudinal evidence that depressive symptoms contribute to cognitive decline in SCD above other known factors. Addressing depressive symptoms may be pivotal in slowing further cognitive deterioration in older adults with SCD.